# Nevirapine plasma concentration is associated with virologic failure and the emergence of drug-resistant mutations among HIV patients in Kenya: A cross sectional study

**DOI:** 10.1097/MD.0000000000032346

**Published:** 2022-12-16

**Authors:** Evans Okumu Omondi, Anne Muigai, Musa Otieno Ngayo, Juster Mungiria, Raphael Lihana

**Affiliations:** a Centre for Microbiology Research, Kenya Medical Research Institute, Nairobi, Kenya; b Department of Botany, Jomo Kenyatta University of Agriculture and Technology, Nairobi, Kenya; c Centre for Virus Research, Kenya Medical Research Institute, Nairobi, Kenya.

**Keywords:** HIV drug resistance, nevirapine plasma concentrations, virologic failure

## Abstract

This study aimed to determine the association between the plasma concentration of nevirapine (NVP) and clinical outcomes. In this cross-sectional study, sociodemographic and clinical data were collected from 233 HIV patients receiving NVP-based first-line antiretroviral therapy (ART) regimens in Nairobi, Kenya. The mean age was 41.2 (SD ± 11.9) years. Fifty-four (23.2%) patients had virological failure (>1000 copies/mL), whereas 23 (9.9%) were infected with drug-resistant HIV strains. Eleven patients had nucleoside reverse transcriptase inhibitor resistance mutations, including M184V and T215Y, whereas 22 had non-nucleoside reverse transcriptase inhibitor resistance mutations, including G190A, K103N, V106A, Y181C, A98G, and Y188L. The median NVP plasma concentration was 6180 ng/mL (IQR 4444–8843 ng/mL), with 38 (16.3%) patients having suboptimal NVP plasma levels of <3400 ng/mL. The majority 23 of the 38 (60.5%) patients with NVP C_min_ < 3400 ng/mL were significantly infected with drug-resistant HIV virus (*P *= .001). In the multivariate analysis, the time taken to arrive at the ART clinic (β −11.1, 95% CI −21.2 to −1.1; *P* = .031), higher HIV viral load (β −2008, 95% CI −3370.7 to −645.3; *P* = .004), and the presence of HIV drug resistance mutation (β 3559, 95% CI 2580.8–4537.2; *P* = .0001) were associated with NVP plasma concentration. A significant proportion of patients receiving the NVP-based regimen had supra- and sub-therapeutic plasma concentrations. Higher HIV viral load and the presence of HIV drug-resistant mutations are important factors associated with NVP plasma concentrations.

## 1. Introduction

Human immunodeficiency virus (HIV) treatment remains complex and many factors must be considered for successful clinical outcomes. Antiretroviral therapy (ART) has been instrumental in reducing the burden of HIV.^[1]^ Kenya initiated the World Health Organization test and treatment guidelines in 2015, which significantly increased the utilization and access to ART treatment.^[2]^ By the end of 2021, approximately 74% of adults in Kenya requiring ART received ART, with approximately 68% achieving virologic suppression.^[1]^ In this study, first-line ART contained a backbone of two nucleoside reverse transcriptase inhibitors (NRTIs; stavudine, tenofovir [TDF], zidovudine, and lamivudine [3TC]) and one non-nucleoside reverse transcriptase inhibitor, either efavirenz or nevirapine (NVP).^[3]^

Optimal drug exposure is critical for ART administration. Prolonged treatment with NVP or its suboptimal exposure has been associated with significant risks of virologic failure and the emergence of HIV drug-resistant strains.^[4–6]^ Data show wider interpersonal variations in NVP plasma levels among HIV patients with treatment failure, and a large proportion fails to achieve a therapeutic window.^[7,8]^ Although several reports have linked plasma NVP concentrations with varied treatment outcomes,^[9]^ in many developing nations, Kenya, including therapeutic drug monitoring, has not yet been integrated into HIV management programs.^[8,10]^

Multiple factors contribute to ART treatment outcomes, including high interpatient variability.^[11]^ These factors are partly due to differences in host genetics and drug metabolism, thus supporting the need for therapeutic drug monitoring to boost the clinical management of HIV.^[12]^ Nevirapine is characterized by a prolonged half-life elimination and a low genetic barrier to resistance, hindering its long-term therapeutic efficacy.^[11]^ Therefore, this study was conducted to determine the relationship between patients’ clinical outcomes, including the presence of HIV drug-resistant mutations, and NVP plasma concentration.

## 2. Materials and Methods

### 2.1. Study design and setting

This cross-sectional study conducted between 2018 and 2019 enrolled 233 HIV patients receiving treatment at the Family AIDS Care and Educational Services (FACES), Kenya Medical Research Institute (KEMRI), Kenya. These patients met the following recruitment criteria: consented to participate, aged ≥ 18 years, and received NVP-based first-line antiretroviral (ARV) treatment for the past 12 months. The first-line ART at the time of this study comprised zidovudine, abacavir, TDF, stavudine, 3TC, and efavirenz or NVP (NVP). The ART regimen formulation and dosing used in this study were per the guidelines of the Ministry of Health, National AIDS, and sexually transmitted infection Control Program.^[3]^

### 2.2. Sample size

The sample size was calculated using the formula described by Lemashow et al,^[13]^ based on population proportion estimation with specified relative precision. The alpha (α) was set at 0.05, relative precision (ε) at 0.20, and the prevalence of individuals on first-line ART was assumed to have drug-related toxicities as a result of abnormally high plasma concentrations of 18%.^[14]^ In total, 233 patients were recruited to achieve 0.95 power.

### 2.3. Data collection

Patient information related to this study was obtained through detailed face-to-face interviews. Approximately 8 mL of blood samples were collected 5 to 8 hours post ARV uptake into three blood tubes as follows: ethylenediaminetetraacetic acid anticoagulant tubes for immunological testing. Serum separation tubes for clinical chemistry, lithium heparin tubes for HIV viral load, and NVP plasma level quantification. The samples were stored at −80°C after collection and analysis. The biological samples were processed at the KEMRI-University of Washington Nairobi laboratory. A sample (whole blood and plasma) aliquot was sent to the Chemistry Laboratory of Jomo Kenyatta University for NVP plasma level validation.

#### 2.3.1. ART drug adherence.

Adherence to ARVs was measured by reviewing the patient medical cards, pharmacy refill data and medical records as described in our previous study Ngayo et al.^[15]^ Adherence was measured based on dose compliance during the 30 days preceding the latest refill. The quantity of dose pills at refill was counted and reconciled against the dose counts dispensed at last refill. Additionally, pill count data were obtained from patient cards for the four months preceding the study period. Nonadherence was determined as the percentage of overdue dose at refill, averaged over a four-month period and used to assign adherence as good (≤5% dose skipped) while as poor (>15% dose skipped).

### 2.4. Quantification of NVP plasma concentrations

#### 2.4.1. Solutions.

Nevirapine (purity: 99.0%) and the internal standard, nevirapine D4 (purity: 99.3%), were purchased from Alsachim (Strasbourg, France). A 200 μg/mL NVP stock solution was diluted with 50% methanol in water to a concentration range of 752.27 and 121,333.33 ng/mL. The internal standard was diluted with 50% methanol to obtain a working solution of 100 ng/mL. Then, 20 µL of the working standard and 20 µL of IS were further diluted in 200 µL of drug-free human plasma to prepare six plasma calibrators at a 10-fold dilution.

#### 2.4.2. Viral inactivation.

The method for viral inactivation has been described in our previous publications.^[15,16]^ Briefly, a mixture of each plasma sample (50 µL) and internal standard (5 µL) in a 1.5 mL Eppendorf tube was heated for 10 minutes at 65°C and then cooled at room temperature for 10 minutes. One hundred microliters (100 µL) of cold methanol (−20°C) was then added and held cold for 10 minutes and subsequently centrifuged at 20,000 g at 20°C for 8 minutes, and the supernatant was collected in a clean 1.5 ml Eppendorf tube. Ammonium acetate buffer (850 L, pH 3.00) was added to the supernatant and briefly centrifuged. The samples were inactive and safe for further processing in the inorganic laboratory.

#### 2.4.3. Quantification of NVP.

The NVP plasma concentrations were quantified using a tandem quadrupole mass spectrometer (LC/MS/MS) designed for ultra-high performance: Xevo TQ-S (Waters Corporation, Massachusetts) as described by Reddy et al^[17]^ modified in our previous publication.^[15,16]^ Plasma samples were extracted using Bond Elut C18 cartridges, according to the manufacturer’s instructions (Agilent Technologies, California). The eluents were completely evaporated using Thermo Scientific Reacti-Vap evaporators (Thermo Fisher Scientific Inc., California) at 37°C for 30 minutes. This was then reconstituted using 100 µL of equal parts 1:1 acetonitrile and water, vortexed briefly, transferred into 50 mL capped vials, and placed into Xevo TQ-S (Waters Corporation) for quantification. Approximately 1 µL of the sample was injected automatically into the LC/MS/MS instrument and quantified within 5 minutes. NVP plasma concentrations were categorized as <3400 ng/mL (below the therapeutic range), 3400 to 6000 ng/mL (therapeutic range), or >6000 ng/mL (above the therapeutic range).^[18,19]^

#### 2.4.4. Blood chemistry.

Cluster of differentiation 4 (CD4) cell counts were measured using a FACSCount TM flow cytometer (BD Biosciences, San Jose, CA), whereas HIV-1 RNA was measured using a Generic HIV Viral Load^®^ (Biocentric, Bandol, France). Assays were performed according to the manufacturer’s instructions.

#### 2.4.5. HIV drug-resistant genotyping.

The presence of HIV drug-resistant mutations was tested using an in-house genotypic method as previously described by Lehman et al.^[20]^ Resistance mutations were identified using Stanford University and International AIDS Society-USA website interpretation algorithms.

### 2.5. Data analysis

All the data were subjected to descriptive data analyses. Frequencies and percentages were used to present sociodemographic data. Steady-state NVP plasma concentrations were not normally distributed according to the Shapiro–Wilk test; hence, the Kruskal–Wallis test, Dunn’s test, and quantile regression analysis were used to evaluate the association with NVP plasma concentrations at the 5% significance level. All statistical analyses were performed using STATA v. 13 (StataCorp LP, Texas).

## 3. Results

### 3.1. Participants characteristics

A total of 233 adult participants receiving a first-line ART regimen were evaluated, with a mean age (SD) of 41.1 (11.9) years. There were 144 (61.8%) females, 85 (36.5%) with a tertiary level of education, 148 (63.5%) were Bantus, 122 (52.4%) were unemployed, 193 (82.8%) were married, and 135 (57.9%) were currently living with their children. There were 105 (45.1%) participants who consumed alcohol, and only seven (3%) abused other drugs. The mean (SD) age of sexual debut was 17.6 (2.3) years, and 167 (71.7%) had the age of sexual debut of < 18 years. The mean (SD) for body mass index was 25.9 (4.9) kg/m^2^, with an average time taken to the ARV clinic of 83.4 (SD ± 47.1) minutes, and an average transportation cost to ARV clinic of 231.1 (SD ± 176.5) Ksh. The average duration of living with HIV was 6.4 (SD ± 5.7) years, with a majority of 160 (68.7%) of the participants taking ARV between 1 and 5 years, while 165 (70.8%) had changed their previous ARV regimen. There were 149 (63.9%) of the participant who had poor ART adherence rate.

The mean (SD) CD4 count for the participants was 418.7 (357.5) cells/μL, with 162 (69.5%) having CD4 < 500 cells/μL. The mean (SD) viral load count for the participants was 17345.1 (89165.7) cells/mL with 179 (76.8%) of the participants being virally suppressed (<1000 cells/mL). Of the 233 participants enrolled, 23 (9.9%) harbored drug-resistant virus at the time of sampling, 26 (11.2%) and 52 (22.3%) had alanine aminotransferase and aspartate aminotransferase levels, respectively, and 67 (28.8%) had anemia (Table 1).

**Table 1:** Summary of patient demographics and clinical characteristics of patients included in the model.

Variables	Frequency	Percentage
Age group		
Mean (±SD)	41.1 (±11.9)	
20–30	40	17.2
31–40	73	31.3
41–50	68	29.2
51–60	40	17.2
>61	12	5.2
Gender		
Male	89	38.2
Female	144	61.8
Education level		
Primary	70	30
Secondary	75	32.2
Tertiary	85	36.5
Non-formal	3	1.3
Ethnicity		
Bantu	148	63.5
Nilotes	2	0.9
Cushite	83	35.6
Occupation		
Formal employment	71	30.5
Self-employment	40	17.2
Unemployed	122	52.4
Ever been married		
Yes	193	82.8
No	40	17.2
Alcohol use		
Yes	105	45.1
No	128	54.9
Drug use		
Yes	7	3
No	226	97
Age of sexual debut (yr)		
Mean (±SD)	17.6 (±2.3)	
* * <18	167	71.7
* * >19	66	28.3
Body mass index (kg/m^2^)		
Mean (±SD)	25.9 (±4.9)	
* * <18.5 (Underweight)	106	45.5
* * 18.5–24.9 (Normal weight)	79	33.9
* * 25–29.9 (Overweight)	3	1.3
* * ≥30 (Obese)	45	19.3
Travel duration to ARV clinic (min)		
Mean (±SD)	83.4 (±47.1)	
* * 1–30	26	11.2
* * 31–60	98	42.1
* * >61	109	46.8
Fare to ARV clinic (Ksh)		
Mean (±SD)	231.1 (±176.5)	
1–250	172	73.82
251–500	51	21.89
>501	10	4.29
Hospital admission		
Yes	6	2.6
No	227	97.4
Duration with HIV (yr)		
1–5	139	59.7
6–10	37	15.9
>11	57	24.4
Duration post ART initiation (yr)		
1–5	160	68.7
6–10	38	16.3
>11	35	15
Changed ARV		
Yes	165	70.8
No	68	29.2
ART adherence		
Good	84	36.1
Poor	149	63.9
Skin rash		
Yes	6	2.6
No	227	97.4
CD4+ (cell/μL)		
Mean (±SD)	418.7 (±357.5)	
* * 1–500	162	69.53
* * >501	71	30.47
HIV viral load (cells/mL)		
Mean (±SD)	17345.1 (±89165.7)	
* * 1–1000	179	76.82
* * >1001	54	23.18
HIV drug resistant mutation		
Yes	23	9.9
No	210	90.1
ALT (U/L)		
<56	207	88.8
≥56	26	11.2
AST (U/L)		
<40	181	77.7
≥40	52	22.3
HB (g/dL)		
<13	67	28.8
≥13	166	71.2

ALT = alanine aminotransferase, ART = antiretroviral therapy, AST = aspartate aminotransferase, ARV = antiretroviral, CD4 = cluster of differentiation 4, HIV = human immunodeficiency virus, HB = hemoglobin, SD = standard deviation.

### 3.2. Drug resistance mutation across the study population

Approximately 23 patients (9.9%) were infected with drug-resistant HIV. G190ASEQ (3.9%) was the most common non-nucleoside reverse transcriptase inhibitor mutation, followed by K103NS 7 (3.1%), V106AM (1.3%), Y181 CIV, and V108IV, each occurring in 2 (0.9%) participants. The most common NRTI mutation was M184V, which occurred in 10 (4.3%) patients. Of the two patients who harbored TAMs mutations, all had T215Y mutations among those who were on 3TC/NVP/TDF treatment. The majority 23 of the 38 (60.5%) patients with NVP C_min_ < 3400 ng/mL were significantly infected with drug-resistant HIV virus, compared to 2 of the 69 (2.9%) patients with NVP C_min_ between 3400 and 6000 ng/mL or 2 of the 126 (1.3%) patients with NVP C_min_ > 6000 ng/mL (*P *= .001).

### 3.3. NVP plasma concentration

The mean NVP plasma (SD±) titer was 7165.1 ± 4544.3 ng/mL ranging between 450 to 44207 ng/mL. The Shapiro–Wilk W test showed that the distribution of the NVP plasma levels was regular among the study participants (Shapiro–Wilk W = 0.81072; V = 32.3; *P* = .0001). The majority 126 (54.1%) of the participants had NVP plasma levels of > 6000 ng/mL, which are considered levels for durable viral suppression. There were 69 (29.6%) participants with NVP levels of < 3400 ng/mL considered levels for poor viral suppression and the least 38 (16.3%) had NVP levels between 3400 and 6000 ng/mL considered levels for viral mutant selection windows.

Participants with NVP levels of > 6000 ng/mL had significantly lower age of sexual debut 83(65.9%) as compared to participants with NVP levels between 3400 and 6000 ng/mL or <3400 ng/mL (*P* = .041). Participants with suboptimal NVP levels of < 3400 ng/mL had lower CD4 count 1 to 500 cell/µL compared to participants with NVP levels between 3400 and 6000 ng/mL or <6000 ng/mL (*P* = .022). Participants with suboptimal NVP levels of <3400 ng/mL were infected with virus with various resistant mutations than participants with NVP levels between 3400 and 6000 ng/mL or <6000 ng/mL (*P* = .0001). Participants with suboptimal NVP plasma levels of <3400 ng/mL had significantly higher viral load >1001 cells/mL than participants with NVP levels between 3400 and 6000 ng/mL or <6000 ng/mL (*P* = .0001). A trend was observed regarding participants with poor ART adherence with a higher percentage of them having lower NVP plasma level <3400 ng/mL when compared to participants with NVP levels = 3400 to 6000 ng/mL and NVP levels > 6000 ng/mL (76.3% vs 63.8% vs 60.3%, respectively; *P* = .202). There was no significant statistical difference observed regarding occurrence of adverse outcome such as skin rash, hemoglobin count, aspartate aminotransferase count, and alanine aminotransferase levels in participants with NVP plasma levels > 6000 ng/mL when compared to participants with lower NVP plasma levels (Table 2).

**Table 2:** Variation in NVP plasma levels across patient demographics and clinical variables.

Variables	NVP level < 3400 ng/mL 69 (29.6%)	NVP level = 3000–6000 ng/mL 38 (16.3%)	NVP level > 6000 ng/mL 126 (54.1%)	*P* value
Age group				
20–30	8 (21.1)	16 (23.2)	16 (12.7)	
31–40	6 (15.8)	25 (36.2)	42 (33.3)	
41–50	15 (39.5)	17 (24.6)	36 (28.6)	.153
51–60	6 (15.8)	9 (13)	25 (19.8)	
>61	3 (7.9)	2 (2.9)	7 (5.6)	
Gender				
Male	11 (28.9)	26 (37.7)	52 (41.3)	.398
Female	27 (71.1)	43 (62.3)	74 (58.7)	
Education level				
Primary	12 (31.6)	23 (33.3)	35 (27.8)	
Secondary	9 (23.7)	22 (31.9)	44 (34.9)	.809
Tertiary	17 (44.7)	23 (33.3)	45 (35.7)	
Non-formal	0	1 (1.5)	2 (1.6)	
Ethnicity				
Bantu	20 (52.6)	46 (66.7)	82 (65.1)	
Nilotes	17 (44.7)	23 (33.3)	43 (34.1)	.36
Cushite	1 (2.6)	0.0	1 (0.8)	
Occupation				
Formal employment	13 (34.2)	15 (21.7)	43 (34.1)	
Self-employment	6 (15.8)	17 (24.6)	17 (24.6)	.217
Unemployed	19 (50)	37 (53.6)	66 (52.4)	
Ever been married				
Yes	31 (81.6)	53 (76.8)	109 (86.5)	.202
No	7 (18.4)	16 (23.2)	17 (13.5)	
Alcohol use				
Yes	18 (47.4)	27 (39.1)	60 (47.6)	.492
No	20 (52.6)	42 (60.9)	66 (52.4)	
Drug use				
Yes	0	2 (2.9)	5 (3.9)	.677
No	38 (100)	67 (97.1)	121 (96.1)	
Age of sexual debut (yr)				
<18	27 (71.1)	57 (82.6)	83 (65.9)	**.041**
>19	11 (28.9)	12 (17.4)	43 (34.1)	
Body mass index (kg/m^2^)				
<18.5 (Underweight)	19 (50)	31 (44.9)	56 (44.4)	
18.5–24.9 (Normal weight)	10 (26.3)	21 (30.4)	48 (38.1)	.482
25–29.9 (Overweight)	1 (2.6)	0	2 (1.6)	
≥30 (Obese)	8 (21.1)	17 (24.6)	20 (15.9)	
Travel duration to ARV clinic (min)				
1–30	3 (7.9)	7 (10.1)	16 (12.7)	
31–60	20 (52.6)	25 (36.2)	53 (42.1)	.524
>61	15 (39.5)	37 (53.6)	57 (45.2)	
Fare to ARV clinic (Ksh)				
1–250	28 (73.7)	49 (71.1)	95 (75.4)	
251–500	9 (23.7)	16 (23.2)	26 (20.6)	.916
>501	1 (2.6)	4 (5.8)	5 (3.9)	
Hospital admission				
Yes	2 (5.3)	2 (2.9)	2 (1.6)	.308
No	36 (94.7)	67 (97.1)	124 (98.4)	
Duration with HIV (yr)				
1–5	25 (65.8)	42 (60.9)	72 (57.1)	
6–10	3 (7.9)	9 (13.1)	25 (19.8)	.466
>11	10 (26.3)	18 (26.1)	29 (23.1)	
Duration post ART initiation (yr)				
1–5	26 (68.4)	47 (68.1)	87 (69.1)	
6–10	6 (15.8)	11 (15.9)	21 (16.7)	.996
>11	6 (15.8)	11 (15.9)	18 (14.3)	
Changed ARV				
Yes	14 (36.8)	19 (27.5)	35 (27.8)	.545
No	24 (63.2)	50 (72.5)	91 (72.2)	
ART adherence				
Good	29 (76.3)	44 (63.8)	76 (60.3)	.202
Poor	9 (23.7)	25 (36.2)	50 (39.7)	
Skin rash				
Yes	2 (5.3)	1 (1.5)	3 (2.4)	.545
No	36 (94.7)	68 (98.5)	123 (97.6)	
CD4+ (cell/μL)				
1–500	33 (86.8)	43 (62.3)	86 (68.3)	**.022**
>501	5 (13.2)	26 (37.7)	40 (31.8)	
HIV viral load (cells/mL)				
1–1000	15 (39.5)	57 (82.6)	107 (84.9)	**.0001**
>1001	23 (60.5)	12 (17.4)	19 (15.1)	
HIV drug resistant mutation				
Yes	19 (50)	2 (2.9)	2 (1.6)	**.0001**
No	19 (50)	67 (97.1)	124 (98.4)	
ALT (U/L)				
<56	36 (94.7)	59 (85.5)	112 (88.9)	.38
≥56	2 (5.3)	10 (14.5)	14 (11.1)	
AST (U/L)				
<40	31 (81.6)	52 (75.4)	98 (77.8)	.787
≥40	7 (18.4)	17 (24.6)	28 (22.2)	
HB (g/dL)				
<13	15 (39.5)	20 (28.9)	32 (25.4)	.233
≥13	23 (60.5)	49 (71.1)	94 (74.6)	

ALT = alanine aminotransferase, ART = antiretroviral therapy, AST = aspartate aminotransferase, ARV = antiretroviral, CD4 = cluster of differentiation 4, HIV = human immunodeficiency virus, HB = hemoglobin, NVP = nevirapine.

### 3.4. Regression analysis

In the multivariate regression analysis, factors that remained associated with NVP plasma concentrations included the time taken to arrive at the ART clinic (β −11.1, 95% CI −21.2 to −1.1; *P = *.031), higher HIV viral load (β −2008, 95% CI −3370.7 to −645.3; *P* = .004), and the presence of HIV drug resistance mutation (β 3559, 95% CI 2580.8–4537.2; *P* = .0001) (Table 3).

**Table 3:** Regression analysis between NVP plasma concentrations and clinical outcomes.

Clinical outcomes	Univariate analysis				Multivariate analysis			
	Unadjusted β	(95% CI)		*P* value	Unadjusted β	(95% CI)		*P* value
Age group	377	−88.9	842.9	.112	171.8	−400.2	743.8	.555
Gender	−553	−1631	525	.313	−360	−1798.1	1078.1	.622
Education level	297	−161	755	.203	325.6	−352.8	1004	.345
Ethnicity	−169.5	−746.7	407.7	.563	−252.6	−814	308.8	.376
Occupation	−249.5	−775.5	276.5	.351	−158.8	−840.6	523	.647
Religion	−754	−4524.5	3016.5	.694	−294.6	−3710.7	3121.5	.865
Ever been married	667	−707.2	2041.2	.34	792.6	−468.5	2053.7	.217
Living with children	−553	−1653	547.0	.323	−173.97	−2158	1810.1	.863
Alcohol use	381	−441	1203	.362	571.6	−239.9	1383.1	.166
Drug use	443	−900.2	1786.2	.516	−123.6	−4468	4220.8	.955
Age of sexual debut (yr)	892	326.3	1457.7	**.002**	1285	−683.1	3253.7	.2
Number of lifetime sexual partners	99	−4	202	.059	−803.8	−2508.3	900.7	.354
BMI (kg/m^2^)	−89.7	−466	287	.639	−147.0	−918.8	624.8	.708
Types of stable food stuff	22	−135	179	.783	27.4	−226.2	281	.832
Access to staple food	1013	−1349	3375	.399	2309	224.2	4393.8	**.03**
Missed staple food	686	19	1353	**.044**	−901.4	−3738.2	1935.4	.532
No of times missed staple food	477	108	846	**.012**	940.1	−494	2374.2	.198
Meal preparation	267	−539	1073	.515	237.8	−570	1045.6	.562
Porridge consumption	−22	−799	755	.956	−64.6	−927.7	798.6	.883
Travel duration to ARV clinic (min)	−489	−1527.6	549.6	.355	−362	−1299.8	575.9	.448
Fare to ARV clinic (Ksh)	−168	−1133.2	797.2	.732	−279.3	−1839.2	1280.5	.724
Hospital admission	−963	−3689.5	1763.5	.487	−997.1	−4285.1	2291	.551
Duration with HIV (yr)	−41	−764.5	682.5	.911	447.9	−308.9	1204.6	.245
Duration post ART initiation (yr)	−166	−670.9	338.9	.518	−439	−1485.6	607.6	.409
Changed ARV	−280	−1105.5	545.5	.505	−568.7	−2058.7	921.4	.453
Current ARV type	460	−351	1271	.265	490.7	−380.9	1362.3	.245
ART adherence	460	−213.8	1133.8	.18	−145.4	−1600.5	1309.7	.844
Skin rash	−88	−3987	3811	.965	−838.2	−4496.3	2819.9	.652
CD4+ (cell/μL)	446	−473.7	1365.7	.34	0.3	−1.4	2.1	.709
HIV viral load (cells/mL)	−2082	−3245	−919	**.001**	−2008.0	−3371	−645.3	**.004**
HIV drug resistant mutation	3868	3109.1	4626.9	**.0001**	3857.1	2871.2	4843	**.0001**
ALT (U/L)	−22	−1029.9	985.9	.966	−300.1	−3519.9	2919.6	.854
AST (U/L)	−41	−917.1	835.1	.927	−276.6	−2195.1	1642	.777
HB (g/dL)	555	−657.6	1767.6	.368	−315	−2418.1	1788.1	.709

ALT = alanine aminotransferase, ART = antiretroviral therapy, AST = aspartate aminotransferase, BMI = body mass index, ARV = antiretroviral, CD4 = cluster of differentiation 4, HIV = human immunodeficiency virus, HB = hemoglobin, NVP = nevirapine.

## 4. Discussion

The integration of ART into Kenya for the management of HIV infection has significantly reduced the mortality and morbidity of patients with HIV. The standard ART regimen in Kenya is restricted to a limited number of combination options, and the inclusion of new regimens is often slow.^[3]^ In the current study, NVP was a widely prescribed component of highly active antiretroviral therapy used alongside other ART drugs to maximize viral suppression.^[3]^ NVP was an extensively prescribed antiretroviral drug in developing countries mainly because of its efficacy, availability, low cost, and ability to prevent vertical HIV transmission. However, a higher incidence of rashes, hepatotoxicity and low genetic barrier to resistance are fundamental factors that restrict the use of NVP. This study investigated the association between clinical outcomes, including HIV drug resistance and virological outcomes, and plasma NVP levels.

In this study, men living with HIV were significantly less likely to be on treatment than women, which reflected the current ART treatment trend in Kenya, with 80% of women and 65% of men accessing ART treatment.^[1]^ Furthermore, the majority of study participants (82.8%) were aged ≥ 31 years, implying that ART coverage among the youth population might still be a challenge in Kenya. The NAAC reports that initiating and continuing treatment is particularly problematic in adolescents and young people.^[21]^ In 2019, only 25% of HIV-infected adolescents received ART, of whom 66% were virally suppressed.^[21]^

This study reported NVP plasma concentrations ranging from 450 to 44207 ng/mL. There were 38 (16.3%) participants with NVP levels <3400 ng/mL who were considered to have poor viral suppression. Other studies have reported varied outcomes, with Shapaya^[22]^ reported fewer (6.2%) patients with sub-therapeutic NVP levels. Oluka et al^[23]^ reported even fewer patients had suboptimal NVP levels. Giacomelli et al,^[24]^ reported that 5.6% of patients had subtherapeutic NVP plasma concentrations. In contrast, Gopalan et al,^[19]^ reported a sub-therapeutic trough plasma NVP concentration in 65% of the children sampled in India.

The prevalence and types of drug-resistant HIV strain infecting patients in this study were consistent with previous reports that showed that during long-term ART treatment, the frequency of specific HIV drug-resistant mutations conferring resistance to NVP (K103N, Y181C, and G190A) were dynamic.^[25]^ The study further showed that the frequency of quasispecies with K103N decreased over time, whereas the proportion of Y181C and G190A increased from 0% to nearly 100%. In the current study, the majority of patients with various drug-resistant mutations had NVP levels < 3400 ng/mL, indicating poor viral suppression. This is consistent with a study by Vardhanabhuti et al,^[25]^ which showed that women of African ancestry with HIV-resistance mutations had a lower median time to NVP IC_50_ than Indian women. In contrast, Oluka et al,^[23]^ reported no association between HIV drug resistance and plasma NVP levels in Kenyan women. Cumulatively, these results indicate a dynamic relationship between plasma NVP levels and the occurrence of drug-resistance mutations. Nonetheless, maintaining an optimal plasma concentration of NVP is critical because a single point mutation at a specific position on the *pol* gene in the HIV-1 genome may confer high-level resistance to NVP.^[26]^ Studies have indicated that a trough plasma NVP concentration (C_*trough*_) of/ = 3 μg/mL is associated with an increased risk of treatment failure by promoting the evolution of drug-resistant viral variants.^[27]^

The relationship between the NVP concentration and virologic responses has been explored in various studies. The study by Duong et al,^[18]^ showed that patients with virologic success had higher NVP plasma concentrations than those with virologic failure (mean ± SD, 4.55 ± 2.08 vs 2.57 ± 1.64 µg/mL, *P* = .003). The INCAS trial showed that NVP plasma concentration ranging between 3.45 and 3.88 µg/mL at week 12 was predictive of virologic success after 52 weeks of therapy.^[28]^ In our study, patients with virologic failure (HIV viral load > 1001 cells/mL) had lower NVP plasma levels (<3400 ng/mL) than patients with NVP plasma concentrations > 3400 ng/mL. Our results are consistent with previous results obtained from clinical trials on Caucasians, Africans, and Chinese; higher NVP trough concentrations were associated with a lower rate of virologic failure.^[26]^ In a Chinese study, the median C_trough_ levels among Chinese patients with partial and complete responses after 6 months of treatment were significantly higher than those among patients with viral failure.^[26]^ In contrast, a previous study in Kenya by Oluka et al,^[23]^ showed no association between plasma NVP levels and viral suppression.

Although adherence was not associated with NVP plasma concentrations in this study, ART adherence is generally recognized as an essential component of the successful management of HIV/AIDS.^[29]^ Studies have consistently shown strong associations between poor ART adherence and adverse clinical outcomes, including patient mortality and disease progression, as measured by CD4 cell count, viral load, and the development of drug resistance.^[30]^ Despite mounting evidence on the benefits of ART adherence, suboptimal adherence remains common in adults and children.^[31]^ This association has been attributed to an unstable steady state in the NVP levels due to poor adherence. This can be further explained by the negative correlation coefficient, whereas the number of days missing to take a dose increased, the plasma drug levels decreased. Different adherence measurement methods (including prescribing practices, ART stockouts, promptness of refill pickup, self-report, and pill counts) have yielded varied results regarding treatment outcomes. In this study, we used patient medical cards, pharmacy refill data and medical records adherence measurement method that can face accuracy challenges especially if the ARV pills are not swallowed or taken in the presence of healthcare giver.^[32]^

Other studies measuring factors associated with NVP plasma levels have identified other independent factors that we did not either measure or find to be significant in this study, including duration of therapy,^[33]^ education level (the higher the education level, the higher the NVP plasma levels attributed to better compliance), and ARV adherence with the level of education.^[34]^ Smoking has been associated with enzyme induction, causing higher metabolism, and hence, reduced plasma NVP levels. In contrast, alcohol uptake is associated with higher plasma NVP levels, which is attributed to the reduced metabolism of NVP due to reduced metabolic enzyme concentrations due to reduced liver function.^[35]^ The previous uptake of the efavirenz-based regimen is associated with higher NVP levels given that the two drugs are metabolized by the same enzymes; hence, the reason for changing from efavirenz might have been due to reduced metabolism, which also affects NVP.^[22]^ The CD4 nadir level has also been shown to influence the NVP plasma level.^[36]^ Other factors include the presence of skin rash, co-treatment with other infections such as tuberculosis, meningitis, malaria, social challenges, and HIV-related disclosures.^[37]^

## 5. Conclusions

This study reported highly heterogeneous NVP plasma concentrations, with a significant proportion of patients receiving the NVP-based regimen had supra- and sub-therapeutic plasma concentrations. Higher HIV viral load and the presence of HIV drug-resistant mutations are important factors associated with NVP plasma concentrations.

## Acknowledgments

The authors would like to thank the study patients enrolled in the FACES-KEMRI care and treatment program as well as the technical staff at KEMRI. We wish to acknowledge Director General KEMRI for allowing us to publish this work.

## Author contributions

**Conceptualization:** Evans Okumu Omondi, Anne Muigai, Musa Otieno Ngayo.

**Formal analysis:** Juster Mungiria, Raphael Lihana.

**Funding acquisition:** Musa Otieno Ngayo.

**Methodology:** Evans Okumu Omondi, Anne Muigai.

**Supervision:** Anne Muigai, Raphael Lihana.

**Validation:** Evans Okumu Omondi, Musa Otieno Ngayo, Juster Mungiria.

**Writing – original draft:** Musa Otieno Ngayo.

**Writing – review & editing:** Evans Okumu Omondi, Anne Muigai, Juster Mungiria, Raphael Lihana.
